# Anisotropic Structural Response of NdGaO_3_ Single Crystals to 147 MeV Kr-Ion Irradiation

**DOI:** 10.3390/ma19184022

**Published:** 2026-09-21

**Authors:** Zhakyp T. Karipbayev, Miramgul S. Tokasheva, Aizat Bakytkyzy, Amangeldy M. Zhunusbekov, Askhat Kakimov, Marina Konuhova, Sofija Konuhova, Vladislavs Bezrukovs, Anatoli I. Popov

**Affiliations:** 1 Department of Technical Physics, Institute of Physics and Technology, L.N. Gumilyov Eurasian National University, Kazhymukan Str. 13, Astana 010008, Kazakhstan; tokashevamiramgul@gmail.com (M.S.T.); zhunusbekov_am@enu.kz (A.M.Z.); kakimov_ab_3@enu.kz (A.K.); marina.konuhova@cfi.lu.lv (M.K.); 2 Research Institute of Functional Materials, Nanotechnologies and Computer Modeling, L.N. Gumilyov Eurasian National University, Kazhymukan Str. 13, Astana 010008, Kazakhstan; aizat.bakytkyzy.1@gmail.com; 3 Institute of Solid State Physics, University of Latvia, Kengaraga 8, LV-1063 Riga, Latvia; sofija.konuhova@cfi.lu.lv; 4 Engineering Research Institute, Ventspils International Radio Astronomy Centre, Ventspils University of Applied Sciences, LV-3601 Ventspils, Latvia; vladislavsb@venta.lv

**Keywords:** NdGaO_3_ single crystal, swift heavy-ion irradiation, krypton ions, X-ray diffraction, lattice strain, radiation-induced disorder, orientation-dependent damage, SRIM simulation

## Abstract

NdGaO_3_ is an orthorhombic perovskite substrate whose response to swift heavy ions remains insufficiently understood. Here, (001)- and (110)-oriented single-crystal wafers were irradiated with 147 MeV ^84^Kr^15+^ ions to 1 × 10^13^ ions/cm^2^ and examined by symmetric Cu Kα X-ray diffraction. Peak shifts were evaluated using a two-parameter strain/sample-displacement model; PDXL was used for phase identification and diagnostic whole-pattern fitting, while SRIM and NIST attenuation data were used to compare the ion range with the absorption-weighted X-ray depth. Irradiation strongly reduced and broadened the principal reflections and enhanced diffuse scattering near 2θ ≈ 30°. Corrected normal strains were 0.30% for (001) and 1.20% for (110). Reflection-specific apparent microstrains were 0.29–0.61% and 0.69–1.31%, respectively. A broad component already present in the Pristine (110) profile increased in fitted area by approximately 63% after irradiation. Full orthorhombic-cell and Williamson–Hall outputs from unconstrained powder-profile fitting are mathematically underdetermined for these highly oriented wafers and were not used quantitatively. Attenuation calculations establish strong overlap between the probed depth and the SRIM-defined modified region, although depth gradients in crystallinity prevent unique assignment of residual Bragg intensity. The results demonstrate pronounced anisotropic disorder without additional crystalline phases.

## 1. Introduction

Neodymium gallate (NdGaO_3_) is a rare-earth gallate with a distorted GdFeO_3_-type perovskite structure. At room temperature, it crystallizes in the orthorhombic Pbnm setting of space group No. 62 (equivalent to the alternative Pnma setting), with four formula units per unit cell [1,2,3,4,5,6]. The framework consists of corner-sharing GaO_6_ octahedra, and the departure from the ideal cubic perovskite structure is governed mainly by cooperative octahedral tilting and displacements of Nd ions within the interoctahedral cavities [1,7,8]. These distortions give rise to pronounced crystallographic anisotropy and influence the thermal, optical, dielectric, and lattice-dynamic properties of the material [7,8,9,10,11,12,13,14].

NdGaO_3_ is widely used as a single-crystal substrate for the epitaxial growth of high-temperature superconductors and other functional oxides, including manganites, nickelates, ferroelectrics, and oxide heterostructures [15,16,17,18,19,20,21,22]. Its technological importance is associated with the availability of high-quality oriented crystals, favorable lattice matching with many perovskite films, and the possibility of obtaining chemically controlled and atomically smooth (001)- and (110)-oriented surfaces [6,7,8]. Surface termination is particularly important because it affects nucleation, interfacial stoichiometry, strain accommodation, and electronic transport in epitaxial heterostructures [18,19,20,21]. Consequently, accurate knowledge of the substrate structure and lattice parameters is essential for evaluating lattice mismatch and strain in coherently grown layers [6].

The room-temperature lattice parameters reported for NdGaO_3_ vary slightly among diffraction studies because the derived values can be affected by sample alignment, instrumental geometry, crystal stoichiometry, and structural defects [1,6,9]. Using a high-resolution modified Bond method, Schmidbauer et al. determined the absolute lattice parameters as a = 5.428410(54) Å, b = 5.498407(55) Å, and c = 7.708878(95) Å [6]. These values provide a particularly reliable reference for strain analysis in single crystals and epitaxial films. For comparison with routine phase-identification measurements, the ICDD PDF card 00-069-0263 lists the orthorhombic Pbnm phase with a = 5.42554 Å, b = 5.49173 Å, c = 7.70441 Å, and V = 229.55 Å^3^ [23]. The difference between database and high-precision values illustrates why the selected reference dataset and possible systematic angular errors must be considered when small irradiation-induced lattice changes are evaluated.

The physical properties of NdGaO_3_ are strongly direction dependent. Anisotropic thermal expansion has been observed along the orthorhombic axes [9,14,16], while the sound velocity and thermal conductivity also reflect the distorted crystal framework [10]. Optical investigations have shown that NdGaO_3_ possesses anisotropic refractive and dielectric behavior [11,12,13]. In particular, generalized spectroscopic ellipsometry performed on (001)-, (101)-, and (110)-oriented single crystals established the complex dielectric functions along the a, b, and c directions over the photon-energy range of 0.73–9.0 eV [11]. The lowest band-to-band transition energies were determined as 6.46(6), 6.29(5), and 6.77(7) eV for polarization along the a, b, and c axes, respectively [11]. These results identify NdGaO_3_ as an ultrawide-bandgap oxide and demonstrate that crystallographic orientation must be considered when interpreting its optical response [11]. NdGaO_3_ is generally considered thermally stable over a wide temperature range, with no pronounced structural discontinuities. Nevertheless, Berkstresser et al. [24] reported a second-order phase transition at approximately 1220 K using differential thermal analysis (DTA). Furthermore, Savytskii et al. [25,26] observed a thermal anomaly near 200 K in NdGaO_3_ single crystals, evidenced by anomalies in the dielectric loss tangent, magnetic susceptibility, and thermal expansion coefficient. To clarify the crystal symmetry and investigate the structural anomaly near 200 K, IR, Raman and even temperature-dependent polarized Raman spectroscopy was performed on a NdGaO_3_ single crystal [8,27]. The interpretation of the spectra is particularly challenging because NdGaO_3_ adopts a strongly distorted orthorhombic perovskite structure (*Pbnm*), in which cooperative rotations and tilts of the GaO_6_ octahedra substantially increase the number of Raman-active lattice vibrations. Nevertheless, 18 well-resolved phonon modes were successfully identified and assigned to the *Pbnm* symmetry: seven A_1g_, four B_1g_, four B_2g_, and three B_3g_ modes [8]. Table 1 presents a comprehensive overview of the experimental data reported for NdGaO_3_ in the literature [1,2,8,9,12,25,26,27]. By consolidating the fundamental structural, dielectric, and physicochemical characteristics, the table establishes a coherent basis for understanding the interplay between the intrinsic properties of NdGaO_3_ and its behavior in functional and technological applications.

The pronounced anisotropy and high structural quality of NdGaO_3_ also make it a useful model system for investigating radiation-induced modifications in complex oxides. When a swift heavy ion penetrates a solid, a major fraction of its kinetic energy may be deposited through electronic stopping. The subsequent transfer of energy from the excited electronic subsystem to the lattice can generate highly localized structural disorder, residual stress, defect-rich regions, and, when the deposited energy exceeds the relevant damage threshold, latent ion tracks or partial amorphization [28,29,30,31,32,33]. The final damage morphology depends on the electronic energy-loss density, ion velocity, thermal and elastic properties of the target, defect-recovery kinetics, and crystallographic structure [33,34,35].

Despite the extensive literature on the pristine crystal structure, substrate preparation, interfacial behavior, and optical properties of NdGaO_3_ [15,16,17,18,19,20,21], systematic studies of high-energy-ion-induced structural modifications in oriented NdGaO_3_ single crystals remain limited. This lack of data is important because irradiation-induced changes in lattice spacing, microstrain, mosaicity, crystalline fraction, or surface-layer disorder may affect the reliability of NdGaO_3_-based substrates and devices operating in radiation environments. Comparison of different orientations is particularly relevant for an orthorhombic material because the response measured by symmetric X-ray diffraction is tied directly to the lattice planes parallel to the sample surface.

A further methodological issue concerns the interpretation of symmetric θ–2θ scans from oriented single crystals. Whole-pattern fitting and Rietveld-type approaches are most robust when numerous independent reflections are available, whereas a flat oriented crystal generally produces only a restricted family of predominantly symmetric reflections. Under these conditions, simultaneous refinement of all orthorhombic unit-cell parameters is mathematically underdetermined and can yield strongly correlated or nonphysical values. Conversely, calculations based on only one or two peak positions do not use the complete diffraction-profile information and may be sensitive to sample displacement, peak asymmetry, diffuse-scattering tails, and weak-reflection uncertainties. Accordingly, the present analysis assigns distinct roles to the two approaches: selected reflections are used for quantitative orientation-specific lattice metrics, whereas PDXL is retained for phase identification and diagnostic assessment of overall profile evolution.

In this work, the structural response of (001)- and (110)-oriented NdGaO_3_ single crystals irradiated with 147 MeV Kr ions to a fluence of 1 × 10^13^ ions cm^−2^ is investigated. The objectives are to (i) determine the magnitude and anisotropy of irradiation-induced changes in interplanar spacing; (ii) separate directional lattice strain from geometric sample-displacement effects; (iii) estimate the apparent microstrain of the residual crystalline component; (iv) define the valid and invalid uses of PDXL/WPPF outputs for highly oriented wafers; (v) distinguish irradiation-enhanced diffuse scattering from a pre-existing broad background component; and (vi) evaluate the overlap between the absorption-weighted Cu Kα information depth and the SRIM-calculated ion-modified region [36,37,38,39,40].

## 2. Materials and Methods

### 2.1. Samples and Irradiation Conditions

Commercial NdGaO_3_ single-crystal substrates with (001) and (110) surface orientations were supplied by Kinheng Crystal Material (Shanghai) Co., Ltd., China. The starting wafers had a diameter of 1 inch, a thickness of 0.5 mm, and double-side-polished flat surfaces.

**Figure 1:**
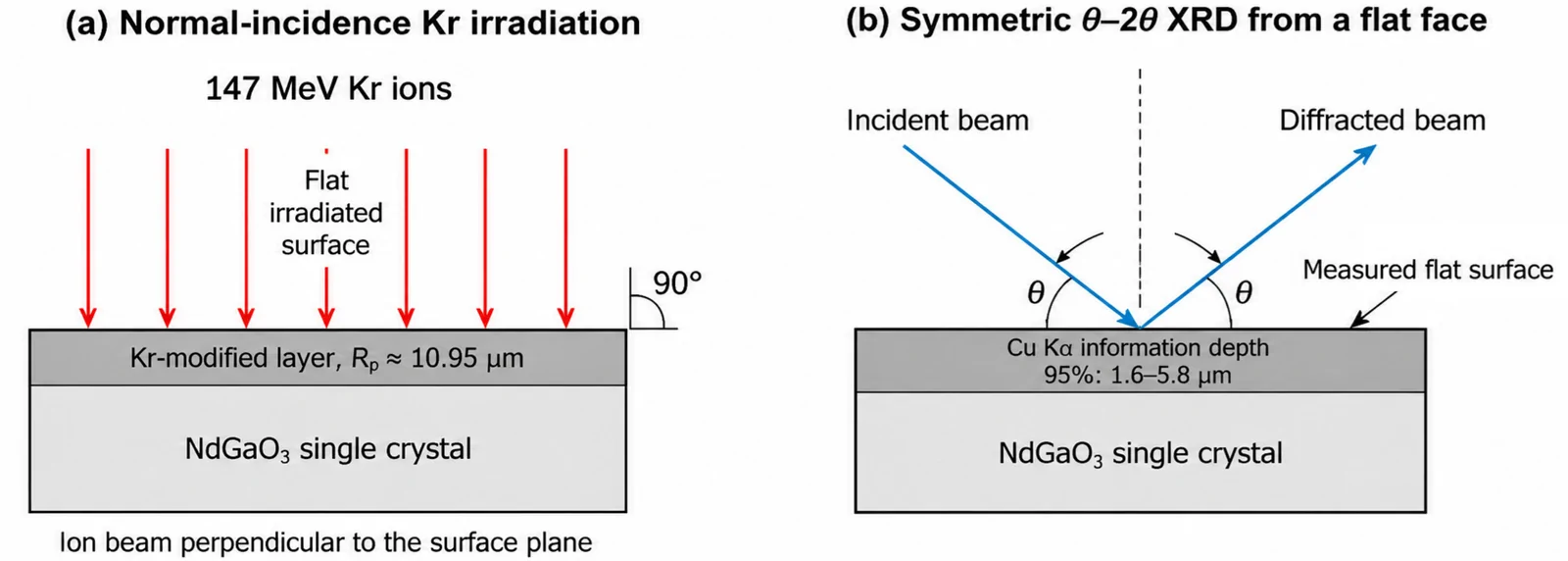
Experimental geometry. (**a**) Normal-incidence irradiation of the flat NdGaO_3_ surface by 147 MeV Kr ions. (**b**) Symmetric θ–2θ XRD measurement from a flat face.

The NdGaO_3_ samples were irradiated with ^84^Kr^15+^ ions at an energy of 1.75 MeV per nucleon, corresponding to a total ion energy of approximately 147 MeV. Irradiation was performed using the DC-60 heavy-ion accelerator at the Institute of Nuclear Physics, Astana, Kazakhstan, which has been extensively employed in our previous studies on radiation-induced pro-cesses and defect evolution in a variety of functional and structural materials [41,42,43,44,45]. The total fluence was 1 × 10^13^ ions cm^−2^, and the ion-beam current density was maintained within the range of 25–30 nA cm^−2^. The beam was incident at 90° to the sample surface, i.e., normal to the irradiated plane (Figure 1a). The total fluence was used as the principal irradiation-dose parameter; no model-based conversion of current density into beam-heating or annealing corrections was introduced.

### 2.2. X-Ray Diffraction Measurements

Structural characterization was performed using a Bruker D6 PHASER X-ray diffractometer (Bruker AXS GmbH, Karlsruhe, Germany) equipped with a Cu-anode source operated at 40 kV and 15 mA. Measurements employed the stored Coupled TwoTheta/Theta method in symmetric θ–2θ geometry, Continuous PSD fast scan mode, and an SSD160 detector operated in one-dimensional pulse-height-analysis (PHA) mode. PHA energy discrimination was used to suppress the Cu K_β_ contribution. The flat specimens were spun about the surface normal during acquisition; for an oriented single crystal, this rotation averages azimuthal surface and mounting inhomogeneity but does not produce powder averaging. The Pristine and Kr-irradiated faces were measured after flipping and remounting each specimen. Acquisition and export parameters are summarized in Table 2.

### 2.3. PDXL Phase Identification and Diagnostic Whole-Pattern Fitting

Phase identification, background modelling, and diagnostic whole-pattern profile fitting were performed using the Rigaku PDXL software package (Rigaku Corporation, Tokyo, Japan). The software accounts for the measured background, peak positions and shapes, the Cu Kα_1_/Kα_2_ doublet, profile asymmetry, and preferred orientation. These capabilities were used to identify crystalline phases and to inspect the global degradation of the diffraction profiles after irradiation.

The full orthorhombic-cell parameters and Williamson–Hall domain-size/microstrain outputs from unconstrained PDXL fitting were not treated as quantitative structural observables. Symmetric scans of the present highly oriented wafers contain only 00L or hh0 specular families and lack the independent asymmetric reflections required to determine a, b, c, and V simultaneously. The numerical instability is directly demonstrated by nonphysical outputs for the narrow Pristine (110) pattern, including a coherent-domain size of 14.21 Å and a Williamson–Hall microstrain of 7.1%. Complete PDXL outputs are retained in Supplementary Table S1 for transparency, but the quantitative strain analysis in the main text is based on direction-specific indexed reflections.

### 2.4. Manual Determination of Direction-Specific Structural Parameters

Interplanar spacings were calculated using Bragg’s law [46]:

(1) $$2d_{hkl}sin\theta =\lambda$$

For an orthorhombic unit cell,

(2) $$\frac{1}{d_{hkl}^{2}}=\frac{h^{2}}{a^{2}}+\frac{k^{2}}{b^{2}}+\frac{l^{2}}{c^{2}}$$

For the (001) orientation, symmetric (00L) reflections directly determine the out-of-plane lattice parameter:

(3) $$c_{out}=l\;d_{00l}.$$

The principal estimate of $c_{out}$ was obtained from the 004 and 006 reflections. The region near $2\theta \approx 23^{\circ }$, where the closely spaced 002 and 110 contributions may overlap, was used only for diagnostic purposes. For the (110) orientation, the primary directly measurable parameter is

(4) $$d_{110}={\left(\frac{1}{a^{2}}+\frac{1}{b^{2}}\right)}^{-1/2}$$

Independent determination of (a) and (b) from the symmetric 110 and 220 reflections is not possible without additional asymmetric reflections. Therefore, the manual analysis of the (110)-oriented samples was restricted to $d_{110}$ and was not intended to reconstruct the complete three-dimensional orthorhombic unit cell independently.

### 2.5. Separation of Lattice Strain and Effective Sample Displacement

When a sample is flipped and remounted, the observed peak shift may contain both a strain-related contribution and a geometric contribution arising from displacement of the measured surface relative to the focusing circle. In the linear approximation,

(5) $$\Delta 2\theta =-2\varepsilon tan\theta -2\frac{h}{R}cos\theta ,$$

where ε is the average normal strain, h is the surface displacement, and R is the goniometer radius; all angular quantities are expressed in radians. For each crystallographic orientation, two available reflections were used to solve the resulting system of equations for ε and the dimensionless parameter h/R.

Because the system contains two equations and two unknowns, the procedure has no internal degrees of freedom. It should therefore not be regarded as an independent metrological calibration, but rather as a physically motivated sensitivity analysis of the effect of sample remounting. The key result is that a non-zero strain contribution remains after inclusion of the geometric term and retains a pronounced orientation dependence.

### 2.6. Bragg-Peak Broadening and Microstrain

In the absence of a separate instrumental standard, the width of the corresponding Pristine reflection was used as an experimental baseline that includes both instrumental broadening and the initial mosaic spread:

(6) $$\beta_{def}=\sqrt{\beta_{irr}^{2}-\beta_{Pristine}^{2}}$$

where the full width at half maximum, β, is expressed in radians. The apparent microstrain associated with an individual reflection was estimated as

(7) $$\varepsilon_{hkl}=\frac{\beta_{def}}{4tan\theta }$$

whereas the equivalent coherent-scattering length scale of the residual Bragg component was calculated as

(8) $$D_{hkl}=\frac{K\lambda }{\beta_{def}cos\theta },$$

with (K = 0.9). These quantities are regarded as apparent values because the contributions of finite domain size, mosaicity, strain gradients, and instrumental broadening were not fully separated.

The manual approach has three fundamental limitations. First, it determines only c_out_ for the (001) orientation and d_110_ for the (110) orientation rather than the complete orthorhombic unit cell. Second, it relies on the positions and local widths of selected reflections and therefore does not account for the complete profile shape, peak asymmetry, or extended diffuse-scattering tails. Third, the analysis is based on only two or three reflections and is consequently sensitive to poor counting statistics, peak overlap, and the selected local background. These limitations are explicitly acknowledged; they justify using PDXL for phase and profile diagnostics, but they do not validate unconstrained PDXL full-cell or Williamson–Hall parameters as quantitative single-crystal constants.

### 2.7. Diffuse Scattering and Short-Range Correlation Length

The broad contribution near 2θ ≈ 30° was not treated as a conventional Bragg reflection. Because a broad maximum is already present in the Pristine (110) profile, the Pristine and Kr-irradiated (110) data were fitted over 25–40° using the same linear-background-plus-Gaussian model. The comparison was based on the fitted center, FWHM, and integrated Gaussian area. For the (001) Pristine profile, the long tails of the intense 00L reflections and periodic PSD-related modulation prevented a stable equivalent baseline separation; consequently, only the characteristic width of the broad component in the Kr-irradiated (001) profile was reported. The magnitude of the scattering vector was calculated as

(9) $$q=\frac{4\pi sin\theta }{\lambda }$$

For a profile width $\beta_{2\theta }$, expressed in radians, the corresponding broadening in reciprocal space was approximated as

(10) $$\Delta q\approx \frac{2\pi cos\theta }{\lambda }\beta_{2\theta },\;\;\xi =\frac{2\pi }{\Delta q}$$

The resulting ξ value is a descriptive correlation length for the broad component and must not be interpreted as a crystallite size, a coherent-domain size of the original single crystal, or a direct measure of amorphous volume fraction.

### 2.8. SRIM Simulations and XRD Information Depth

SRIM-2013.00 calculations were performed for Kr in an Nd–Ga–O target with a density of 7.57 g cm^−3^ and atomic composition Nd:Ga:O = 20:20:60. The supplied file is a SRIM stopping/range table, not a TRIM displacement-damage simulation. Therefore, Quick Kinchin–Pease versus Full Cascade settings, the number of simulated collision histories, and threshold displacement energies for Nd, Ga, and O do not enter the reported S_e_, S_n_, R_p_, or straggling values. The quoted S_e_ = 19.84 keV/nm and S_n_ = 0.05151 keV/nm are the tabulated entrance values at the incident energy of 147 MeV; they are not depth-averaged values or maxima. The available output does not contain a depth-resolved stopping or vacancy-production profile. The principal parameters are summarized in Table 3.

Because no depth-resolved S_e_(z), vacancy, or dpa profile was generated, the SRIM results are used only to establish the incident stopping regime and the characteristic projected range/straggling envelope. No depth-dependent defect concentration is inferred from the single entrance stopping value.

In symmetric θ−2θ geometry, the contribution from a layer located at depth z is attenuated according to

(11) $$I\left(z\right)=I_{0}exp\left(-\frac{2\mu z}{sin\theta }\right)$$

where μ is the linear X-ray attenuation coefficient. The depth containing a fraction *p* of the total diffraction signal is therefore given by

(12) $$z_{p}=-\frac{ln\left(1-p\right)sin\theta }{2\mu }$$

**Figure 2:**
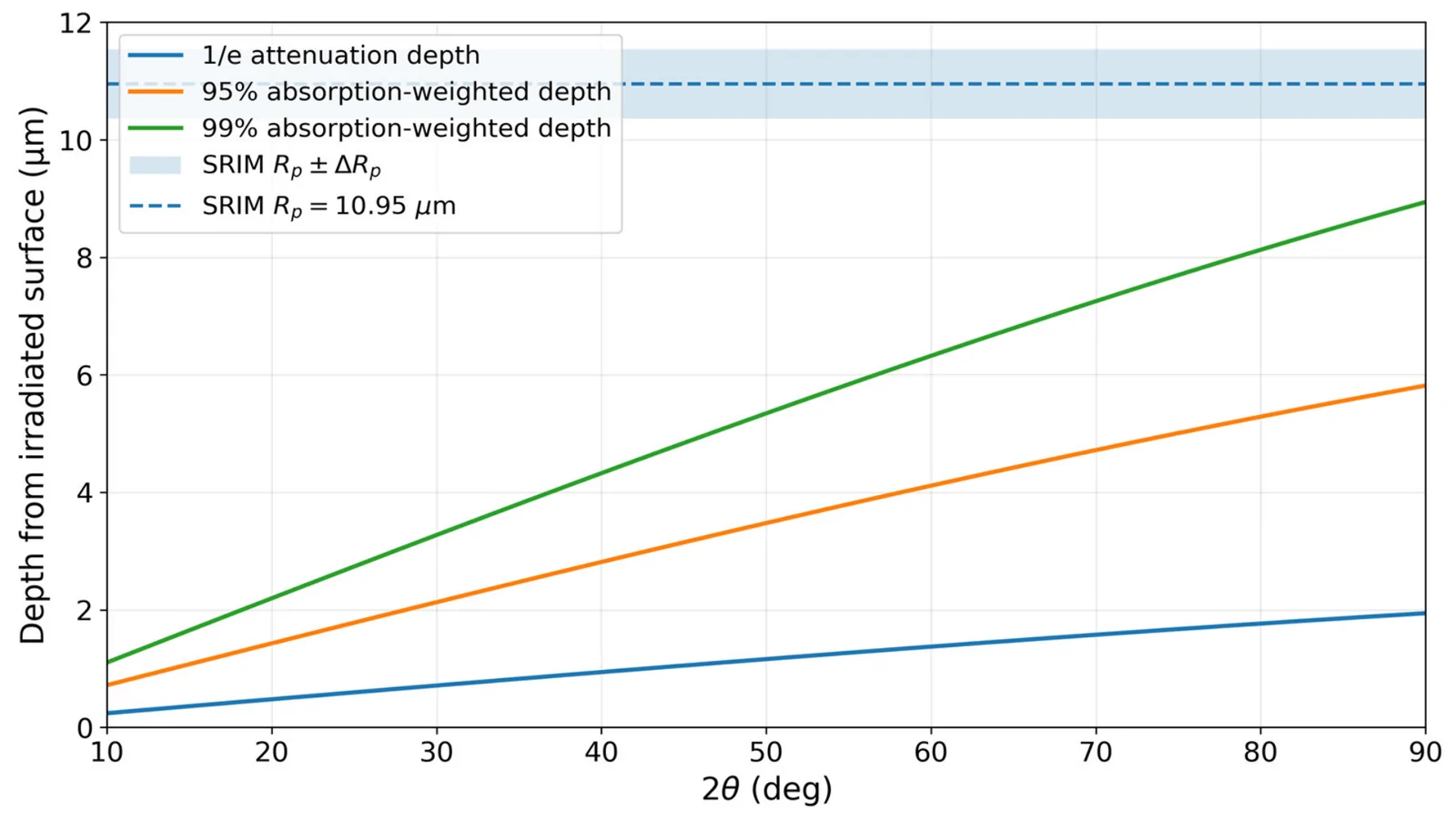
Calculated absorption-weighted Cu Kα XRD information depth in NdGaO_3_ using NIST attenuation coefficients [47], compared with the SRIM projected range and longitudinal straggling of 147 MeV Kr ions [37]. The attenuation curves do not include a depth-dependent crystalline fraction.

The linear attenuation coefficient was calculated using the mixture rule

(13) $$\mu =\rho \sum_{i}w_{i}{\left(\frac{\mu }{\rho }\right)}_{i}$$

where ρ is the density of NdGaO_3_, w_i is the mass fraction of element i, and (μ/ρ)_i is its mass attenuation coefficient. The mass fractions were taken from the stoichiometric composition used in the SRIM target, and the elemental coefficients were interpolated at the Cu Kα photon energy (8.048 keV) from the NIST tables [47]. The complete calculation is shown in Table 4 and Figure 2.

The weighted mass attenuation coefficient is therefore (μ/ρ)_NdGaO3_ = 240.57 cm^2^ g^−1^, and multiplication by ρ = 7.5700 g cm^−3^ gives μ = 1.821 × 10^3^ cm^−1^.

The calculated depths in Table 5 describe absorption weighting for a depth-independent diffracting fraction. They establish that the measurement is strongly surface weighted and that its nominal information depth overlaps the SRIM-defined ion-modified region. They do not, by themselves, uniquely determine the depth origin of the residual Bragg intensity, because the crystalline fraction and strain may vary strongly with depth; a highly disordered surface layer can attenuate the beam while contributing little coherent Bragg scattering.

### 2.9. Bounding Sensitivity Estimate of Geometrical Track Overlap

For sensitivity analysis, the single-impact Poisson model proposed by Gibbons [48] was employed:

(14) $$A=1-exp\left(-\pi r^{2}\Phi \right),$$

where A is the geometrically covered fraction, r is an assumed radius of a modified track core, and Φ is the fluence. Because the latent-track radius in NdGaO_3_ has not been measured directly, the calculations for r = 2–4 nm define only lower-to-upper geometrical overlap scenarios. They are not used to infer an experimental track radius, an amorphous volume fraction, or a depth-dependent damage profile.

## 3. Results

### 3.1. Phase Identification and Initial Structural State

PDXL identified only crystalline NdGaO_3_ in all four datasets; no additional sharp reflections attributable to secondary crystalline phases appeared after irradiation.

The “100% NdGaO_3_” value reported by PDXL represents normalization among the crystalline phases included in the fitting model and must not be interpreted as a measurement of the total crystalline fraction. In particular, this value does not exclude an amorphous or strongly disordered component, which contributes predominantly to diffuse scattering rather than to indexed Bragg reflections.

Owing to the metrically pseudocubic character of orthorhombic NdGaO_3_, the interplanar spacings d_002_ = c/2d and d_110_ = (a^−2^ + b^−2^)^−1/2^ are nearly identical. According to the reference data, the corresponding CuKα_1_ peak positions are 2θ = 23.070° for 002 and 23.025° for 110, while the higher-order 004 and 220 reflections occur at 47.147° and 47.051°, respectively [10,23]. Therefore, the low-angle reflections cannot be assigned solely from their 2θ positions. In the present work, however, the 002 and 110 peaks were not treated as overlapping components of the same diffraction profile because they were recorded from separate, supplier-specified (001)- and (110)-oriented single-crystal substrates. In symmetric θ–2θ geometry, the scattering vector is parallel to the surface normal; consequently, the specular reflection families are 00L for the (001) surface and hh0 for the (110) surface. The assignments were additionally verified by the corresponding higher-order reflection sequences and by the agreement of the calculated $c_{\mathrm{out}}\;$ and d_110_ values with the reference data. Thus, the near coincidence of the peak positions reflects the pseudocubic metric of NdGaO_3_ rather than mixed orientation or incorrect indexing.

For the Pristine (001) surface, the manual calculation based on the 004 and 006 reflections yielded c_out_ = 7.6995 Å, differing from the reference-card value by only −0.07%. For the Pristine (110) surface, d_110_ = 3.8556 Å was obtained, compared with d_110_,PDF ≈ 3.860 Å. This agreement confirms the reflection assignment, the crystallographic orientation of the measured flat surfaces, and the high degree of long-range order in the initial state.

### 3.2. XRD Patterns of the (001)- and (110)-Oriented Samples

**Figure 3:**
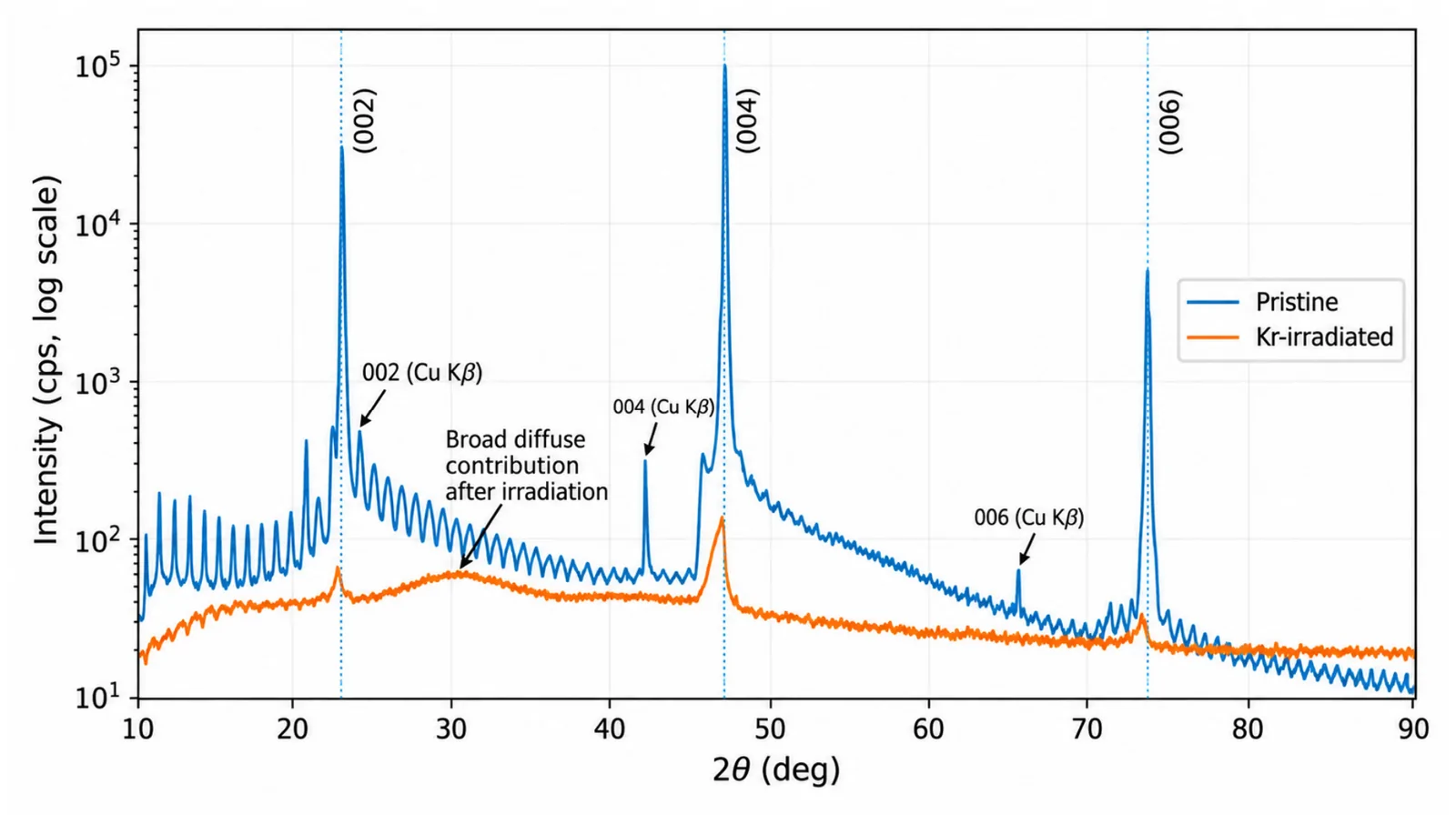
Symmetric θ−2θ XRD patterns of (001)-oriented NdGaO_3_ measured from the Pristine and Kr-irradiated surfaces. The principal 002, 004, and 006 specular reflections are indicated. Weak secondary peaks near $2\theta \approx 20.92^{\circ },42.44^{\circ },$ and $\;65.70^{\circ }$ are attributed to residual Cu K_β_ contributions from the corresponding intense 002, 004, and 006 reflections and are not associated with secondary crystalline phases.

The Pristine (001) pattern is dominated by intense and narrow 002, 004, and 006 reflections. Following Kr-ion irradiation, the principal Bragg intensities decrease by several orders of magnitude, the residual reflections broaden, and an additional broad diffuse contribution becomes apparent near 2θ ≈ 30° (Figure 3). The persistence of weak 004 and 006 reflections demonstrate that some oriented coherent scattering remains after irradiation, although its exact depth distribution cannot be established from attenuation alone.

**Figure 4:**
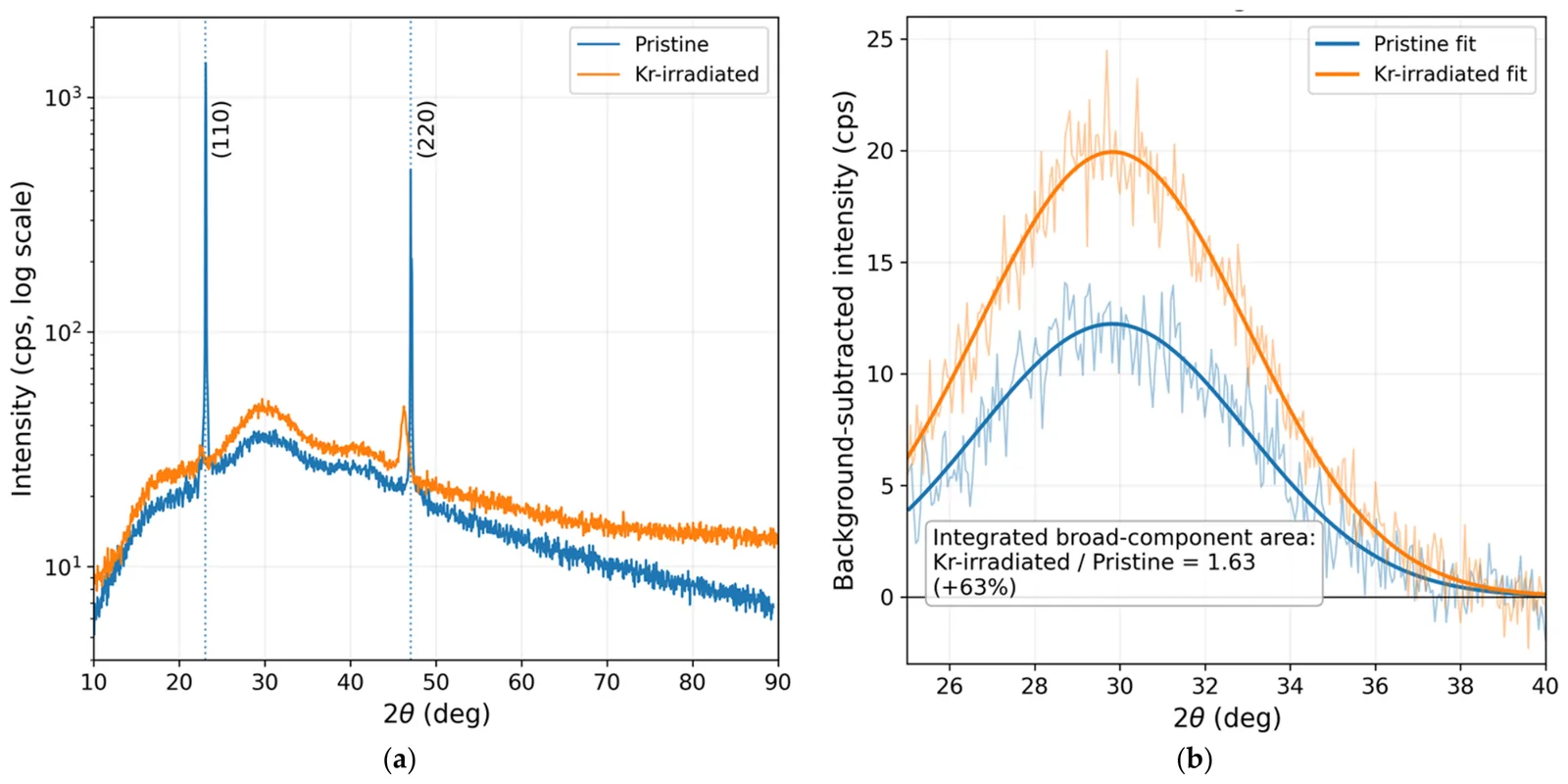
NdGaO_3_ (110) profiles. (**a**) Symmetric θ–2θ patterns for the Pristine and Kr-irradiated surfaces. (**b**) Identical linear-background-plus-Gaussian fits of the broad component over 25–40°. Blue curves correspond to the Pristine sample and orange curves to the Kr-irradiated sample. The lighter thin curves represent the background-subtracted experimental data, whereas the darker smooth curves represent the corresponding fitted Gaussian components. The fitted integrated area increases by approximately 63% after irradiation, demonstrating irradiation-enhanced diffuse scattering rather than the formation of a wholly new halo.

For the Pristine (110) surface, narrow 110 and 220 reflections dominate the diffraction pattern. A broad component centered near 30° is present before irradiation and is enhanced in the Kr-irradiated profile; therefore, it is not described as a wholly radiation-created halo. Under the identical linear-background-plus-Gaussian treatment, the integrated broad-component area increases by approximately 63%, while its fitted center and width remain nearly unchanged (Figure 4). The residual 110 and 220 reflections become markedly weaker and broader after irradiation.

The Pristine (001) scan also contains fine, nearly periodic intensity modulations and extended tails around the very intense single-crystal reflections. Their approximately regular angular spacing (about 0.85–0.95°), lack of correspondence with the allowed NdGaO_3_ reference reflections, concentration in the highest-intensity Pristine profile, and the use of Continuous PSD fast acquisition indicate an instrument/profile origin consistent with detector-channel stitching or related PSD response rather than additional crystalline phases. These features were not indexed and were excluded from all local Bragg-peak and diffuse-component fits. Because no empty-holder or detector-calibration scan was acquired, this assignment is stated as an instrumental-consistency interpretation rather than a uniquely proven mechanism.

Several weak narrow features in the Pristine (001) diffraction pattern were additionally examined to clarify their origin. The sharp peaks observed at $2\theta \approx 20.92^{\circ },42.44^{\circ },$ and $\;65.70^{\circ }\;$ are quantitatively consistent with residual Cu Kβ  contributions associated with the exceptionally intense specular 002, 004, and 006 reflections, respectively. Using the experimentally measured positions of the corresponding $\mathrm{Cu}\;K\alpha_{1}$ reflections and λ_Kβ_ = 1.39225 Å, the calculated Cu K_β_ positions are approximately $2\theta \approx 20.89^{\circ },42.43^{\circ },\;$ and $\;65.69^{\circ }$, in agreement with the observed secondary maxima within approximately $0.03^{\circ }$. The presence of weak residual Cu K_β_ intensity is consistent with the use of electronic pulse-height analysis (PHA) discrimination rather than a crystal monochromator and becomes particularly evident because of the exceptionally high intensity of the pristine single-crystal reflections. These peaks were therefore not assigned to additional NdGaO_3_ phases and were excluded from all quantitative strain and peak-broadening analyses. In addition to the isolated residual Cu K_β_ peaks discussed above, the Pristine (001) scan exhibits a series of weak quasi-periodic intensity modulations and extended tails surrounding the exceptionally intense 002, 004, and 006 specular reflections. These features were carefully examined but were not assigned to crystallographic reflections. Their approximately regular angular spacing does not follow the non-equidistant reflection positions expected from the orthorhombic NdGaO_3_ reference pattern, and such a dense sequence of off-specular hkl reflections is incompatible with the symmetric θ−2θ geometry of a highly oriented (001) single crystal. Moreover, their amplitude closely follows the intensity envelope of the major specular peaks and becomes strongly suppressed in the much lower-intensity Kr-irradiated pattern. Taken together with the continuous one-dimensional PSD acquisition mode used in the present measurements, these observations indicate that the fine oscillatory modulation and extended tails are predominantly instrumental/profile-response features associated with the exceptionally high count rates of the Pristine single-crystal reflections. Consequently, these structures were not indexed and were excluded from all peak-position, FWHM, strain, and microstrain analyses.

### 3.3. Bragg-Peak Shifts and Separation of Strain and Remounting Contributions

All selected residual reflections shift toward lower diffraction angles after irradiation, corresponding to an increase in the associated interplanar spacings. Because the Pristine and Kr-irradiated surfaces were measured after flipping and remounting the specimens, the raw angular displacement contains both a structural contribution and a geometric sample-displacement contribution. The two-parameter results are summarized in Table 6.

**Figure 5:**
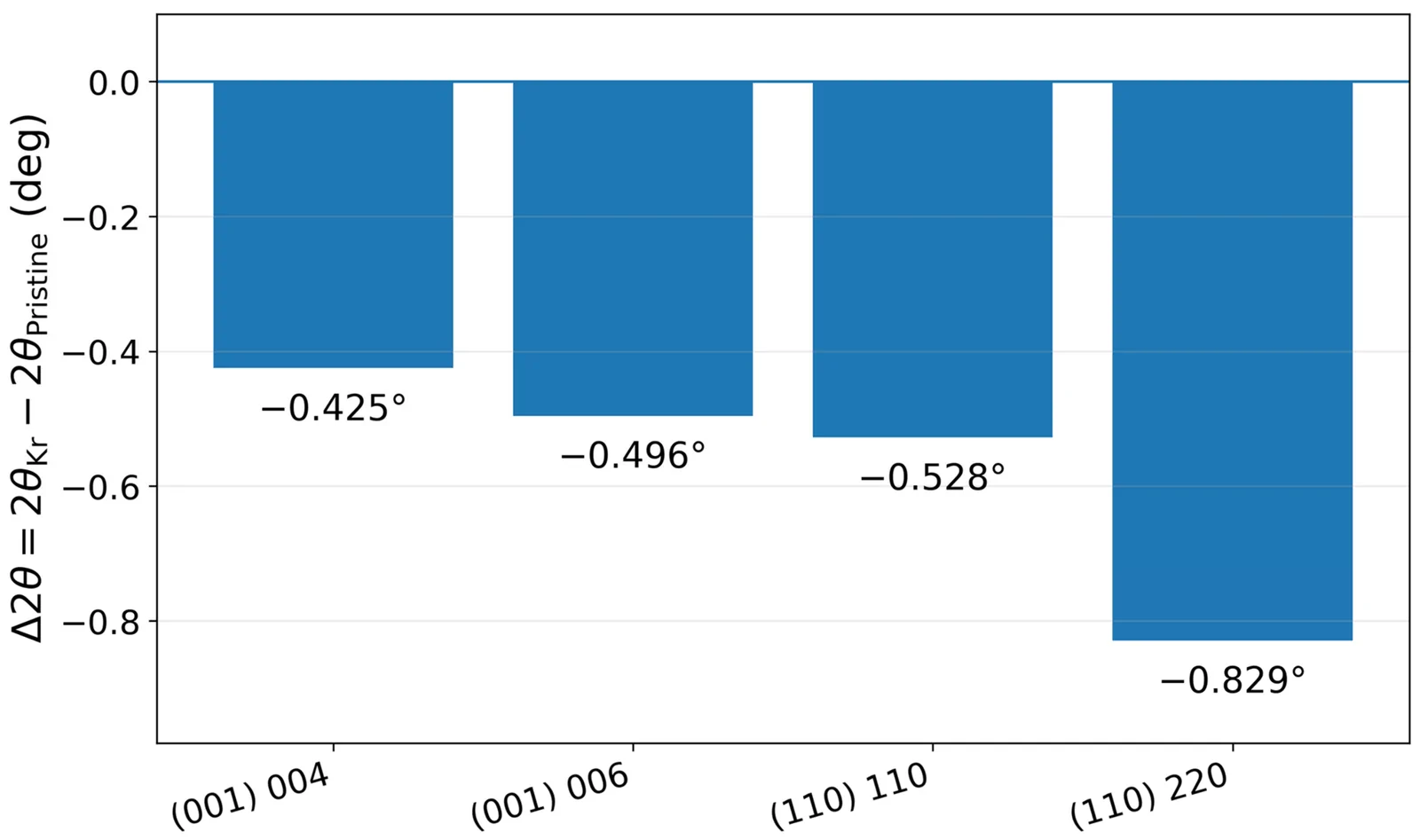
Observed Bragg-peak shifts after irradiation with 147 MeV Kr ions. Negative values denote displacement toward lower diffraction angles.

If the peak shifts were caused solely by an identical sample-height error, their angular dependence would follow the cosθ term in the displacement correction. The experimental reflection pairs do not follow this behavior; most notably, the higher-angle 220 reflection of the (110) specimen shifts more strongly rather than weaklier (Figure 5). After inclusion of both geometric and strain terms, a positive lattice strain remains for both orientations. The corrected strain for (110), approximately 1.20%, is about four times larger than that for (001), approximately 0.30%.

### 3.4. Scope of PDXL Results and Direction-Specific Manual Parameters

PDXL consistently identified the orthorhombic NdGaO_3_ phase and provided a useful diagnostic representation of background and profile evolution. However, the unrestricted full-cell and Williamson–Hall outputs fail internal physical-plausibility checks for the highly oriented specimens. Most clearly, the Pristine (110) pattern, which contains narrow intense Bragg reflections, was assigned D = 14.21 Å and ε_WH_ = 7.1%; these values cannot describe the coherent-domain size or microstrain of a high-quality single crystal.

Compensating changes in the refined a and b parameters can preserve a plausible d_110_ or cell volume while individual constants deviate strongly from accepted NdGaO_3_ values. This is evidence of parameter covariance and mathematical underdetermination, not an integral quantitative measurement of a physically strained three-dimensional unit cell. Accordingly, the complete unconstrained PDXL outputs are reported only in Supplementary Table S1 and are excluded from the quantitative conclusions.

Table 7 therefore compares the direction-specific manual metrics with reference values and explicitly states the restricted role of PDXL. The quantitative structural conclusions rely on c_out_ for (001), d_110_ for (110), and the two-reflection strain/displacement separation. The numerical agreement between the corrected c_out_ and the fitted PDXL c for the irradiated (001) surface is noted only as qualitative consistency and is not treated as an independent validation of the full-cell refinement.

In this sense, manual and automated analyses remain complementary only at different analytical levels: the manual method provides the physically constrained direction-specific observables, whereas PDXL provides phase identification and diagnostic whole-profile visualization.

### 3.5. Broadening of the Residual Bragg Reflections and Apparent Microstrain

**Figure 6:**
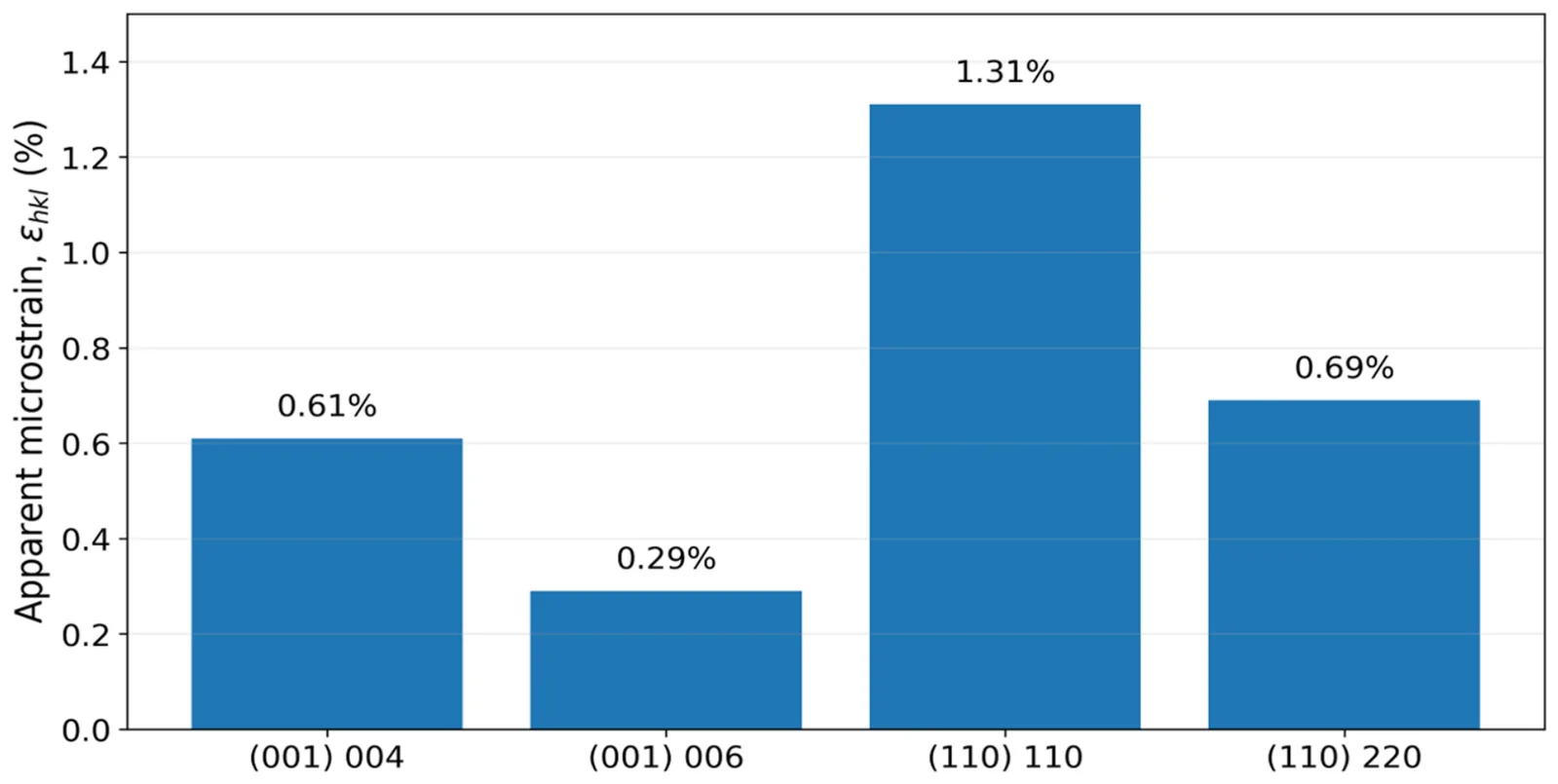
Reflection-specific apparent microstrain estimated from the residual Bragg reflections after quadratic subtraction of the corresponding Pristine peak widths. No PDXL Williamson–Hall values are included because those outputs are underdetermined for the present oriented single crystals.

After quadratic subtraction of the corresponding Pristine peak width, the apparent microstrain was 0.61% from 004 and 0.29% from 006 for the (001) orientation, with associated coherent-scattering length scales of approximately 143 and 199 Å. For the (110) orientation, the values were 1.31% from 110 and 0.69% from 220, with corresponding length scales of approximately 120 and 145 Å (Table 8 and Figure 6). These are reflection-specific apparent quantities: finite coherent length, mosaicity, strain gradients, and residual instrumental effects are not fully separated.

### 3.6. Baseline-Controlled Diffuse Scattering and Short-Range Order

For the Kr-irradiated (001) profile, the broad component is centered at approximately 30.22° with an FWHM of 5.74°, corresponding to a descriptive correlation length ξ ≈ 15.9 Å. A stable equivalent broad-component fit was not extracted from the Pristine (001) profile because the result is strongly coupled to the long tails and periodic PSD-related modulation. For the (110) orientation, identical linear-background-plus-Gaussian fits give centers of 29.82° and 29.83° and FWHM values of 7.46° and 7.45° for the Pristine and Kr-irradiated profiles, respectively. The corresponding integrated Gaussian area increases by a factor of 1.63 after irradiation, while ξ remains approximately 12.3 Å (Table 9). Thus, the principal irradiation effect is an increase in the amplitude of a pre-existing broad component rather than the appearance of a uniquely new halo.

Formal application of the Scherrer equation to this broad component would yield an ångström-scale length similar to the nonphysical PDXL “crystallite-size” outputs. Such a value is not a crystallite size of the single crystal. It is retained only as a reciprocal-space correlation scale, and neither its magnitude nor the broad-component area is converted into an amorphous fraction.

### 3.7. Absorption-Weighted XRD Depth Relative to the Ion-Modified Region

**Figure 7:**
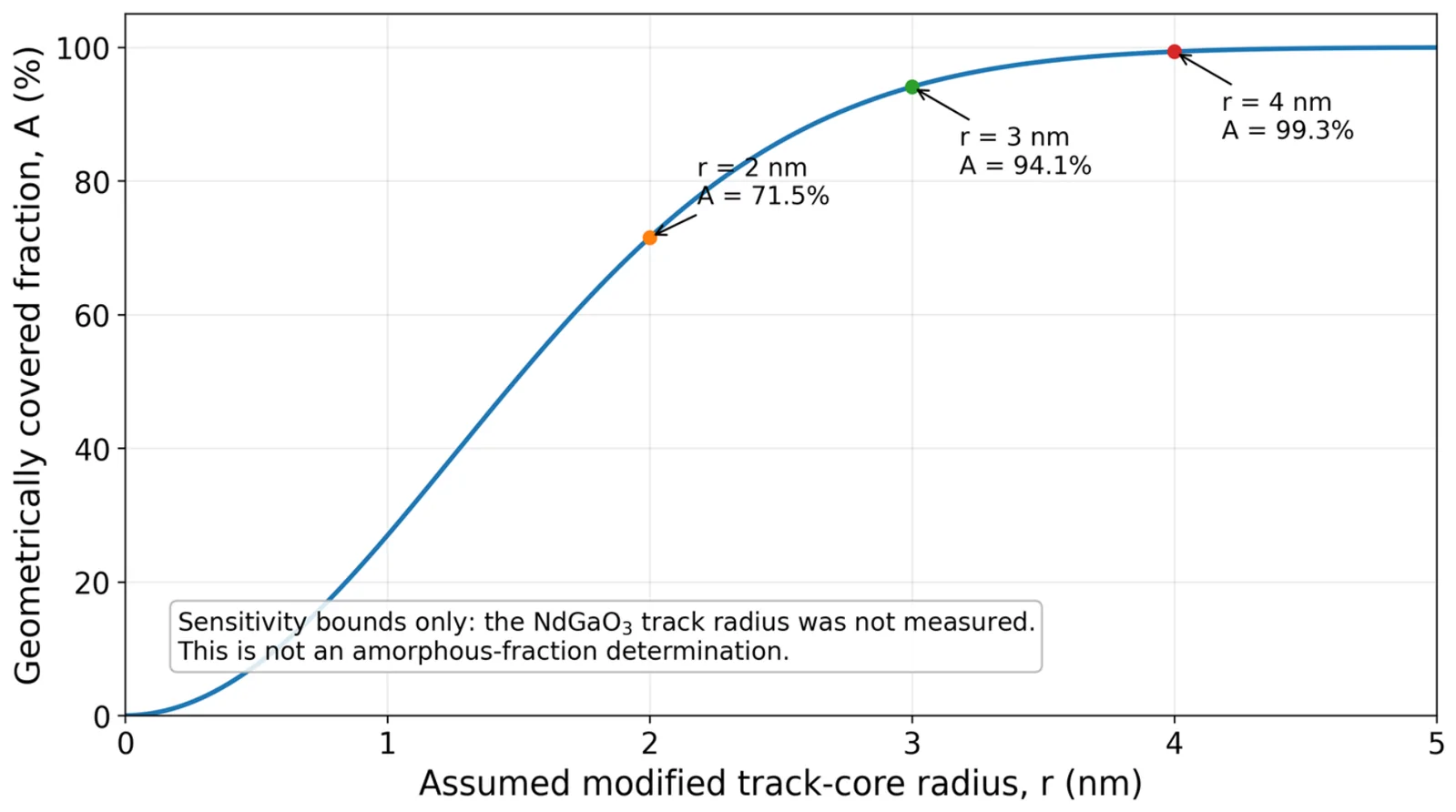
Bounding sensitivity estimate of geometrical track overlap at Φ = 1 × 10^13^ ions cm^−2^ using the single-impact model [48]. The track-core radius was not measured in NdGaO_3_, and the calculation is not an amorphous-fraction determination.

Across the measured angular range, the calculated 95% absorption-weighted depth does not exceed 5.82 μm and the 99% depth does not exceed 8.95 μm, both below the projected Kr range R_p_ = 10.95 μm (Table 10 and Figure 7). This demonstrates that the experiment is strongly weighted toward depths lying within the nominal SRIM range. However, attenuation alone cannot establish the unique origin of the residual Bragg peaks: if the near-surface crystalline fraction is severely reduced, coherent Bragg scattering can be disproportionately weighted toward deeper, less damaged portions of the still ion-modified region. The calculation therefore establishes depth overlap and surface weighting, not an exclusive depth assignment.

### 3.8. Bounding Sensitivity Estimate of Track Overlap

For assumed radii of 2, 3, and 4 nm, the model gives geometrically covered fractions of 71.5%, 94.1%, and 99.3%, respectively. These numbers only bound the overlap expected for hypothetical radii. They are not compared directly with a measured amorphous fraction and do not establish that NdGaO_3_ contains tracks of any particular radius. Direct TEM, SAXS, or another track-sensitive measurement would be required to select among these scenarios.

## 4. Discussion

### 4.1. Adequacy of the Measurement Geometry and Physical Meaning of h/R

Symmetric XRD measurements from flat surfaces are well suited for determining whether an oriented crystalline component remains and for evaluating changes in interplanar spacing along the surface normal. The agreement of the Pristine reflections with the reference card, together with the consecutive 00L and hh0 reflection series, confirms the intended orientations. At the same time, this geometry does not provide enough independent reflections for rigorous three-dimensional refinement of the orthorhombic unit cell.

The fitted values h/R = 2.64 × 10^−3^ for (001) and 2.22 × 10^−3^ for (110) are effective geometric parameters rather than physical thicknesses of a swollen layer. Under the illustrative assumption R = 200 mm, they correspond to h ≈ 0.53 and 0.44 mm, respectively. These displacements are larger than a typical fine alignment error, but they can collectively include differences in specimen seating after flipping, mounting-layer thickness, slight non-parallelism of the opposite faces, and a change in the mean reflecting plane. Because the absolute height was not measured independently for either mounting, h must not be interpreted as radiation-induced swelling. Its role is to remove a common geometric contribution and test whether the orientation-dependent structural trend remains.

After this correction, the strain remains positive, with ε_corr,(110)_ ≈ 1.20% being approximately four times ε_corr,(001)_ ≈ 0.30%. The observed anisotropy therefore cannot be reduced to a single sample-height error.

### 4.2. Distinct Roles and Limitations of Manual Analysis and PDXL

The revised analysis assigns asymmetric roles to the two methods. The manual reflection-specific approach is used quantitatively because the symmetric geometry directly constrains c_out_ for (001) and d_110_ for (110). Its limitations remain substantial: only a few reflections are available, local profile fitting is sensitive to weak-signal statistics and background choice, and the method cannot reconstruct the full strain tensor or complete orthorhombic cell.

PDXL remains valuable for phase identification, Cu Kα_1_/Kα_2_-aware profile representation, and visualization of the global loss of sharp Bragg intensity. It is not, however, a quantitatively valid source of unrestricted a, b, c, V, coherent-domain size, or Williamson–Hall microstrain for the present highly oriented wafers. The extreme Pristine (110) outputs demonstrate that the fitted parameters compensate for one another and for non-Bragg profile contributions.

Consequently, the phrase “effective structural parameters” has been removed as a justification for quantitative use. The complete PDXL numerical outputs are preserved in the Supplementary Materials solely for transparency and reproducibility. The main conclusions are based on indexed peak positions, the explicit sample-displacement correction, and reflection-specific broadening.

The apparent agreement between the corrected manual c_out_ = 7.722 Å and the fitted PDXL c ≈ 7.72 Å for the irradiated (001) surface is treated only as qualitative consistency. It cannot validate the remaining full-cell or Williamson–Hall outputs because the covariance matrix and independent asymmetric reflections required for such validation are unavailable.

### 4.3. Damage Mechanism Under Dominant Electronic Stopping

The entrance stopping-power ratio S_e_/S_n_ ≈ 385 demonstrates that initial energy deposition at the irradiated surface is dominated by electronic rather than nuclear stopping. The combined observations—loss of Bragg intensity, strong broadening of residual reflections, enhanced diffuse scattering, and positive direction-specific strain—are consistent with severe electronic-energy-loss-driven disorder and stressed crystalline regions, as commonly observed for swift heavy ions in complex oxides [28,33,36,49,50,51,52,53].

They do not, on their own, constitute direct microscopy of latent tracks or a quantitative measurement of an amorphous fraction.

The absence of additional crystalline phases does not imply the absence of chemical point defects. Oxygen vacancies, interstitial species, local coordination changes, and rotations of the GaO_6_ octahedra can preserve the average NdGaO_3_ phase assignment without producing separate sharp reflections. Direct identification of such defects would require complementary Raman spectroscopy, XPS/XANES, EPR, or TEM measurements.

### 4.4. Strain Anisotropy and Relation to the Known Lattice Anisotropy

Published data demonstrate strongly anisotropic lattice compliance in NdGaO_3_. High-temperature XRD and synchrotron studies show that expansion along the orthorhombic b direction is substantially weaker than along a and c [9,14]. This intrinsic anisotropy establishes that the lattice does not relax isotropically, but thermal-expansion coefficients cannot be mapped directly onto swift-ion-induced strains because the latter arise from highly localized electronic excitation and defect generation rather than homogeneous heating.

(15) $$\alpha_{d_{110}}=\frac{b^{2}\alpha_{a}+a^{2}\alpha_{b}}{a^{2}+b^{2}}\approx 6.9\times 10^{-6}\;K^{-1}$$

Although the incident electronic stopping is determined primarily by the ion–target combination, the subsequent atomic relaxation occurs within a direction-dependent Pbnm network of tilted GaO_6_ octahedra and inequivalent oxygen sites. Irradiation-induced point defects and locally disordered regions can therefore generate anisotropic displacement and stress fields. The (001) scan mainly probes the c-axis projection, whereas d_110_ couples the responses of the orthorhombic a and b directions. Direction-dependent changes in Ga–O–Ga angles, cooperative octahedral rotations, interstitial/vacancy relaxations, and the projection of oxygen-sublattice displacements onto [001] and [110] provide a physically plausible basis for ε_110_ ≈ 4ε_001_. The difference is interpreted as anisotropic lattice relaxation, not as evidence that four times more electronic energy was deposited in the (110) specimen.

This microscopic interpretation remains a structural hypothesis. Reciprocal-space maps, rocking curves, and asymmetric reflections would be required to determine the complete strain tensor, while polarized Raman spectroscopy and cross-sectional TEM or small-angle scattering would be required to test octahedral-displacement and track-morphology scenarios directly.

### 4.5. Depth Weighting and the Origin of Residual Bragg Scattering

Comparison of the SRIM range with the absorption-weighted X-ray information depth shows substantial overlap between the region sampled by Cu Kα diffraction and the nominal ion-modified layer. Nevertheless, the standard attenuation expression assumes that the diffracting fraction is independent of depth. In reality, the local crystalline fraction, mosaicity, and strain are expected to vary through the damaged region. A highly disordered near-surface zone may contribute strongly to attenuation but weakly to coherent Bragg scattering, thereby shifting the surviving Bragg contribution toward deeper and less damaged portions of the ion-affected layer.

Accordingly, the residual reflections are not assigned exclusively to either a homogeneous damaged surface layer or an unaffected bulk substrate. The defensible conclusion is that the experiment is strongly surface weighted, that its nominal absorption depth lies within the SRIM range, and that the residual Bragg peaks likely integrate a depth-dependent surviving crystalline fraction. The several-orders-of-magnitude intensity loss still demonstrates a major reduction in long-range coherent order, but the depth profile of that reduction cannot be reconstructed from the present θ–2θ data alone.

### 4.6. Experimental and Modelling Limitations

The principal experimental limitations are the absence of an empty-holder/background scan and detector calibration scan, the lack of independently recorded numerical divergence- and anti-scatter-slit openings in the exported metadata, and the availability of only one scan per measured face. Although the same stored method, detector mode, tube settings, and sample spinning were used for all four profiles, these limitations prevent a unique attribution of every broad or periodic profile feature. The periodic modulation in the Pristine (001) scan is therefore excluded from indexing rather than assigned a structural origin.

The structural analysis is further limited by the absence of asymmetric reflections, reciprocal-space maps, an independent instrumental broadening standard, and direct measurement of the specimen height after remounting. The available SRIM file is a stopping/range summary rather than a full depth-resolved cascade calculation, and the Gibbons analysis uses hypothetical track radii. These restrictions are now incorporated explicitly into the interpretation and prevent claims of a complete strain tensor, quantitative amorphous fraction, measured track radius, or uniquely resolved depth origin of the residual Bragg intensity.

## 5. Conclusions

Irradiation of (001)- and (110)-oriented NdGaO_3_ single crystals with 147 MeV ^84^Kr^15+^ ions to 1 × 10^13^ ions cm^−2^ causes a pronounced, orientation-dependent degradation of coherent diffraction. PDXL identifies only the orthorhombic NdGaO_3_ phase, with no additional crystalline products; however, unconstrained reconstruction of the complete orthorhombic cell and Williamson–Hall parameters from the highly oriented θ–2θ profiles is mathematically underdetermined. The resulting full-cell volumes, domain sizes, and PDXL microstrains are therefore not interpreted as quantitative single-crystal observables.

After explicit separation of the strain and remounting contributions, the normal strain is approximately 0.30% for (001) and 1.20% for (110). Reflection-specific apparent microstrains are 0.29–0.61% and 0.69–1.31%, respectively, confirming a stronger structural response along the (110) normal. This anisotropy is attributed to direction-dependent relaxation of defect and stress fields within the tilted GaO_6_ framework rather than to a corresponding difference in the incident electronic stopping. The broad component near 30° is not wholly created by irradiation in the (110) specimen: identical baseline-controlled fits show that it is already present in the Pristine profile and its integrated area increases by approximately 63% after irradiation.

SRIM-2013.00 gives an entrance electronic stopping of 19.84 keV nm^−1^ and a projected range of 10.95 μm. The Cu K_α_ attenuation calculation shows that the experiment is strongly weighted to depths overlapping this ion-modified region. Nevertheless, depth-dependent loss of crystallinity can bias residual Bragg scattering toward deeper, less damaged material, so attenuation alone does not provide a unique depth assignment. The Gibbons calculation is retained strictly as a bounding sensitivity test because the NdGaO_3_ track radius is unmeasured. Overall, the data establish substantial irradiation-induced loss of long-range order and robust crystallographic anisotropy while defining the quantitative limits of both manual and automated diffraction analysis.

## Figures and Tables

**Table 1:** Representative structural and physicochemical properties of NdGaO_3_ reported in the literature.

		Temp	Ref
Lattice parameters Å 0rthorhombic:	*a* = 5.426, *b* = 5.502, *c* = 7.706	RT	[2]
	*a *= 5.4176(2), *b *= 5.4952(2), *c *= 7.6871(3) *a *= 5.4276(1), *b *= 5.49790(9), *c *= 7.7078(1)	100 K 300 K	[1]
	*a *= 5.41842(2); *b *= 5.49534(2); *c*= 7.69311(3)	12 K	[26]
Band gap energy (eV)	3.8	RT	[12]
Raman spectra frequencies (cm^−1^)	94.0; 171.3; 184.8; 205.9; 244.0; 318.1; 355.3; 426.6	83–350	[27]
7A_1g_ (YY, ZZ) 7B_1g_ (XY) 5B_2g_ (XZ) 5B_3g_ (YZ_	104, 148, 217, 297, 344, 414, 474 89, 155, 183, 219, 365, 453 91, 146, 336, 412, 425, 466 90, 171, 183, 206, 355	21 K	[8]
IR spectra frequencies (THz)	92; 119.7; 169; 174; 184.1; 195; 243.6; 274.1; 293.6; 319.6; 427.2; 519; 581;	83–350	[27]
Density	7.57 g/cm^3^	25 °C	
Melting point:	1600 °C to 1605 °C		[9]
C_11_, C_22_, C_33_, C_12_, C_13_, C_23_, C_44_, C_55_, C_66_	326.3; 313.5; 259.6; 155,2; 131.6; 133.2, 122.2; 80.8; 108.0		[9]
*θ*_D_ (K)	513.3		[9]

**Table 2:** XRD acquisition and data-export parameters.

Parameter	Setting/Available Metadata
Diffractometer	Bruker D6 PHASER; Cu anode; 40 kV, 15 mA
Geometry/stored method	Coupled TwoTheta/Theta; symmetric θ–2θ
Scan range	Approximately 2θ = 10–90°
Native exported angular increment	0.05300° for Pristine files; 0.05129° for Kr-irradiated files
Scan mode	Continuous PSD fast
Detector	SSD160_2, 1D mode, pulse-height analysis (PHA)
Cu Kβ suppression	Detector pulse-height discrimination; no separate Kβ peak was included in the analysis
Acquisition time	1.500 s nominal control-program increment; 31.2 s effective TimePerStep recorded in the XY export
Sample spinning	Enabled about the surface normal
Optical settings	Same stored method for all profiles
Repeatability	One available scan per measured face; no independent replicate series

**Table 3:** Irradiation parameters and SRIM-2013.00 stopping/range results [37].

Parameter	Value
Output type	Stopping/range table; no displacement-damage cascade was calculated
Ion	Kr, mass 83.912 amu; incident energy 147 MeV
Experimental fluence	1 × 10^13^ ions cm^−2^
Target composition and density	Nd:Ga:O = 20:20:60 at.%; 7.5700 g cm^−3^
Bragg correction	0.00%
Electronic stopping, S_e_	19.84 keV nm^−1^ at 147 MeV (entrance value)
Nuclear stopping, S_n_	0.05151 keV nm^−1^ at 147 MeV (entrance value)
S_e_/S_n_	≈385
Projected range, R_p_	10.95 μm
Longitudinal/lateral straggling	0.591/0.744 μm
Damage model, ion histories, and E_d_	Not applicable to the stopping/range quantities reported here

**Table 4:** Calculation of the NdGaO_3_ mass and linear attenuation coefficients at the Cu Kα photon energy.

Element	Mass Fraction, w_i_	(μ/ρ)_i_ at 8.048 keV (cm^2^/g)	w_i_(μ/ρ)_i_ (cm^2^/g)
Nd	0.5506	403.15	221.97
Ga	0.2662	62.00	16.50
O	0.1832	11.42	2.09
Total	1.0000	—	240.57

**Table 5:** Calculated absorption-weighted information depth of the Cu Kα diffraction signal in NdGaO_3_.

2θ (deg)	Principal Reflections	z_1_/e (μm)	z_95_% (μm)	z_99_% (μm)
23	002/110	0.55	1.64	2.52
47	004/220	1.10	3.28	5.04
74	006	1.65	4.95	7.61
90	Upper angular limit of the scan	1.94	5.82	8.95

**Table 6:** Bragg-peak positions before and after irradiation and results of the strain–displacement separation.

Orientation, hkl	2θ_Pristine_ → 2θ_Kr_ (deg)	Δ2θ; Raw Δd/d	h/R	Corrected Result
(001), 004	47.202 → 46.776	−0.425°; 0.857%	2.64 × 10^−3^	ε_corr_ = 0.296%; c_out_ = 7.722 Å
(001), 006	73.747 → 73.250	−0.496°; 0.581%	2.64 × 10^−3^	ε_corr_ = 0.296%; c_out_ = 7.722 Å
(110), 110	23.074 → 22.545	−0.528°; 2.312%	2.22 × 10^−3^	ε_corr_ = 1.195%; d_110_ = 3.902 Å
(110), 220	47.054 → 46.225	−0.829°; 1.692%	2.22 × 10^−3^	ε_corr_ = 1.195%; d_110_ = 3.902 Å

**Table 7:** Direction-specific manual lattice metrics, reference comparison, and the restricted analytical role of PDXL.

Sample	Direction-Specific Manual Result	Reference/Corrected Interpretation	Role of PDXL
(001) Pristine	c_out_ = 7.6995 Å	−0.07% relative to ICDD c = 7.70441 Å	Phase identification and diagnostic profile fitting; unconstrained full-cell and WH values not used quantitatively.
(001) Kr-irradiated	c_out,corr_ = 7.722 Å; ε_corr_ = 0.296%	Expansion normal to (001)	Fitted c is qualitatively consistent but not an independent lattice constant because full-cell covariance remains unresolved.
(110) Pristine	d_110_ = 3.8556 Å	Close to d_110_,ref ≈ 3.860 Å	Nonphysical WH and full-cell outputs demonstrate underdetermination and are excluded.
(110) Kr-irradiated	d_110_,_corr_ = 3.902 Å; ε_corr_ = 1.195%	Expansion normal to (110)	Used only for phase/profile diagnostics; no quantitative a, b, c, V, D, or ε_WH conclusion is drawn.

**Table 8:** Reflection-specific apparent microstrain and coherent-scattering length scale of the residual Bragg component.

Orientation/Reflection	Apparent ε_hkl_ (%)	Apparent D_hkl_ (Å)
(001), 004	0.61	≈143
(001), 006	0.29	≈199
(110), 110	1.31	≈120
(110), 220	0.69	≈145

**Table 9:** Baseline-controlled parameters of the broad diffuse-scattering component.

Sample/Profile	Center, 2θ (deg)	FWHM (deg)	Baseline-Controlled Result
(001) Kr-irradiated	30.22	5.74	ξ ≈ 15.9 Å; no stable equivalent Pristine fit
(110) Pristine	29.82	7.46	Relative integrated area = 1.00; ξ ≈ 12.3 Å
(110) Kr-irradiated	29.83	7.45	Relative integrated area = 1.63; ξ ≈ 12.3 Å

**Table 10:** Bounding single-impact Poisson sensitivity estimate of geometrical track overlap at Φ = 1 × 10^13^ ions cm^−2^ [48].

Assumed Track-Core Radius, r (nm)	πr^2^Φ	Geometrically Covered Fraction, A (%)
2	1.257	71.5
3	2.827	94.1
4	5.027	99.3

## Data Availability

The original contributions presented in this study are included in the article. Further inquiries can be directed to the corresponding authors.

## Supplementary Materials

The following supporting information can be downloaded at https://www.mdpi.com/article/10.3390/ma19184022/s1.

## Abbreviations

The following abbreviations are used in this manuscript:

**Abbreviation**	**Definition**
XRD	X-ray diffraction
PDXL	Rigaku powder diffraction analysis software package
WPPF	Whole-powder-pattern fitting
SRIM	Stopping and Range of Ions in Matter
FWHM	Full width at half maximum
WH	Williamson–Hall
TEM	Transmission electron microscopy
SAXS	Small-angle X-ray scattering
NIST	National Institute of Standards and Technology
