# Supplementary material for: Anisotropic Structural Response of NdGaO3 Single Crystals to 147 MeV Kr-Ion Irradiation
Source: Materials (Basel). 2026 Sep 21;19(18):4022. doi: 10.3390/ma19184022 (PMC13608770; doi:10.3390/ma19184022)
Supplement: Supplementary file 1 [file materials-19-04022-s001.zip › materials-4507115-supplementary.pdf]

Supplementary Information

Anisotropic Structural Response of $NdGaO_3$ Single Crystals to 147 MeV Kr-Ion Irradiation

Zhakyp T. Karipbayev, Miramgul S. Tokasheva, Aizat Bakytkyzy, Amangeldy M. Zhunusbekov, Askhat Kakimov, Marina Konuhova, Sofija Konuhova, Vladislavs Berzukovs, and Anatoli I. Popov

S1. Scope of the Supplementary Information

This Supplementary Information file contains the numerical PDXL/WPPF outputs retained for transparency, the baseline-controlled broad-component analysis used to distinguish irradiation-enhanced diffuse scattering from a pre-existing broad background, and archived XRD acquisition records supporting the reproducibility statement in the revised manuscript. **The unconstrained PDXL full-cell and Williamson–Hall outputs are not used as independent quantitative single-crystal observables.**

S2. Complete Unconstrained PDXL/WPPF Outputs

Table S1 reports the numerical values returned by the unconstrained PDXL whole-pattern refinement. These quantities are preserved for transparency and reproducibility. The Pristine (110) outputs D = 14.21 Å and ε_{WH} = 7.1% are incompatible with the sharp single-crystal Bragg peaks and directly demonstrate mathematical underdetermination of the unrestricted powder-profile model for the present specular single-crystal data.

Table S1. Complete unconstrained PDXL/WPPF outputs and their analytical status.

Sample	a (Å)	b (Å)	c (Å)	V (Å^3)	PDXL D (Å)	PDXL WH strain (%)	Analytical status
(001) Pristine	5.503(11)	5.385(10)	7.79(6)	230.9(18)	311(4)	0.17(3)	Full cell underdetermined; a/b correlated. Retained only as software output.
(001) Kr-irradiated	5.944(14)	5.104(11)	7.72(2)	234.3(10)	13.17(4)	4.15(14)	Fitted c is qualitatively consistent with c_{out}; full-cell and WH outputs are not used quantitatively.
(110) Pristine	5.11(3)	5.87(4)	7.68(6)	230(3)	14.21(16)	7.1(3)	Nonphysical D/WH values demonstrate underdetermination; a/b compensate.
(110) Kr-irradiated	5.39(11)	5.93(16)	7.97(9)	255(9)	5.82(2)	6.1(6)	Model-dependent output only; V = 255 Å^3 is not interpreted as true volumetric swelling.

S3. Baseline-Controlled Analysis of the Broad Diffuse-Scattering Component

For the (110) orientation, the Pristine and Kr-irradiated profiles were fitted over 25–40° using the same linear-background-plus-Gaussian model. The fit demonstrates that a broad component is already present before irradiation and that irradiation primarily increases its integrated intensity. The broad component is therefore described as irradiation-enhanced diffuse scattering rather than as a wholly radiation-created amorphous halo. The fitted area is not converted into an amorphous fraction.

Table S2. Baseline-controlled broad-component parameters.

Profile	Fit interval (2θ)	Center (deg)	FWHM (deg)	Integrated Gaussian area (cps·deg)	Relative area	Correlation length ξ (Å)	Interpretation
(001) Kr-irradiated	—	30.22	5.74	—	—	≈15.9	Broad component characterized; no stable equivalent Pristine fit because of intense 00l tails and periodic PSD-related modulation.

Profile	Fit interval (2θ)	Center (deg)	FWHM (deg)	Integrated Gaussian area (cps·deg)	Relative area	Correlation length ξ (Å)	Interpretation
(110) Pristine	25–40°	29.8248	7.458	97.08	1.000	≈12.3	Pre-existing broad component.
(110) Kr-irradiated	25–40°	29.8338	7.451	158.07	1.628	≈12.3	Integrated broad-component area increased by ≈63%.

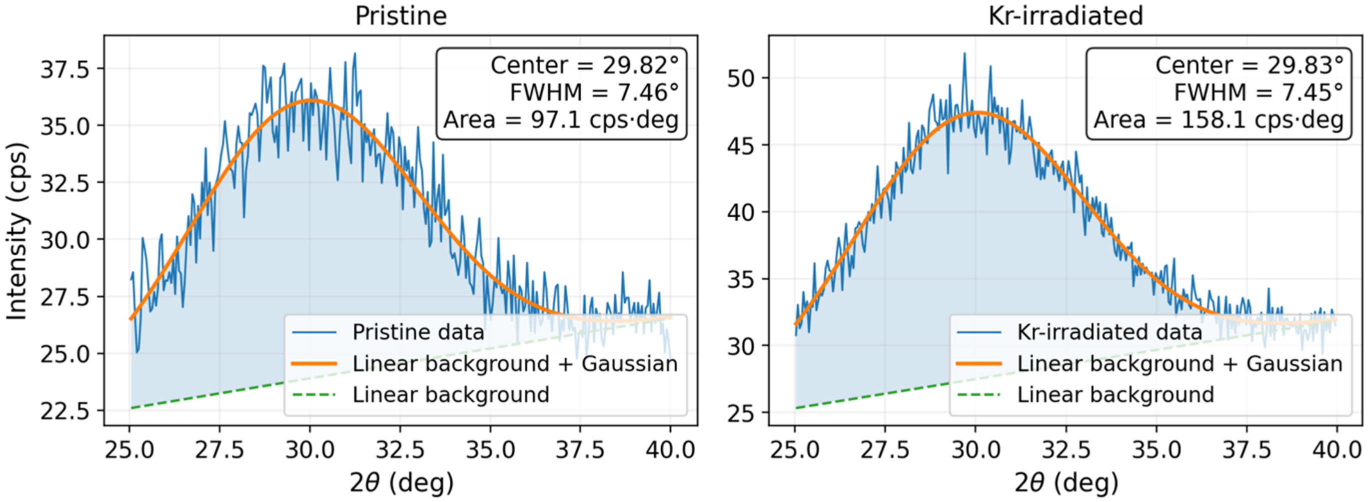

Figure S1. Baseline-controlled fits of the broad component in the $NdGaO_3$ (110) Pristine and Kr-irradiated profiles over 25–40°. The same linear-background-plus-Gaussian model was applied to both profiles; the integrated Gaussian area increases by a factor of 1.63 after irradiation.
